# Action Observation Areas Represent Intentions From Subtle Kinematic Features

**DOI:** 10.1093/cercor/bhy098

**Published:** 2018-05-02

**Authors:** Atesh Koul, Andrea Cavallo, Franco Cauda, Tommaso Costa, Matteo Diano, Massimiliano Pontil, Cristina Becchio

**Affiliations:** 1 Department of Psychology, University of Torino, Torino, Italy; 2 C’MON, Cognition, Motion and Neuroscience Unit, Fondazione Istituto Italiano di Tecnologia, Genova, Italy; 3 GCS-fMRI, Koelliker Hospital and Department of Psychology, University of Torino, Torino, Italy; 4 Focus Lab, Department of Psychology, University of Torino, Torino, Italy; 5 Computational Statistics and Machine Learning, Fondazione Istituto Italiano di Tecnologia, Genova, Italy; 6 Department of Computer Science, University College London, London, UK

**Keywords:** action observation, intentions, kinematics, mirror neurons, MVPA

## Abstract

Mirror neurons have been proposed to underlie humans’ ability to understand others’ actions and intentions. Despite 2 decades of research, however, the exact computational and neuronal mechanisms implied in this ability remain unclear. In the current study, we investigated whether, in the absence of contextual cues, regions considered to be part of the human mirror neuron system represent intention from movement kinematics. A total of 21 participants observed reach-to-grasp movements, performed with either the intention to drink or to pour while undergoing functional magnetic resonance imaging. Multivoxel pattern analysis revealed successful decoding of intentions from distributed patterns of activity in a network of structures comprising the inferior parietal lobule, the superior parietal lobule, the inferior frontal gyrus, and the middle frontal gyrus. Consistent with the proposal that parietal regions play a key role in intention understanding, classifier weights were higher in the inferior parietal region. These results provide the first demonstration that putative mirror neuron regions represent subtle differences in movement kinematics to read the intention of an observed motor act.

## Introduction

How do people so effortlessly detect others’ intentions by simply observing their movements? Mirror neurons have been proposed to be the neural substrate that enables understanding of others’ actions and intentions, by transforming visual information into motor knowledge (Rizzolatti and Craighero 2004). Despite 2 decades of research, however, the exact computational and neuronal mechanisms implied in this visuomotor transformation remain unclear.

One apparent obstacle in converting low-level representations of the movement kinematics to high-level representations of intentions is the supposed multiplicity of mappings between movements and intentions (Jacob and Jeannerod 2005). If you see someone in the street raise his or her hand, is that person hailing a taxi or swatting a wasp? If the same visual kinematics can be caused by different intentions, then any movement-based matching mechanism will fail to get a grip of intentions (Kilner et al. 2007a, 2007b; Gergely and Csibra 2008; Kilner 2011; Clark 2016). This has led some to speculate that it would be impossible for a mirror neuron system driven uniquely by the visual input to correctly encode the intention of an observed action (Jacob and Jeannerod 2005; Kilner 2011; Clark 2016; Hasson and Frith 2016).

However, quantitative behavioral studies are beginning to expose a coextension of kinematics and intentions much deeper than previously thought (Cavallo et al. 2016; see also Ansuini et al. 2015). For example, Cavallo et al. (2016) report that slight variations in movement kinematics convey specificational intention information, that is, information that specifies the intention of the agent in performing a given motor act (Becchio et al. 2017). What is more, observers are sensitive to this information and can use it to discriminate the intention of an observed motor act in the absence of contextual information (Cavallo et al. 2016). This suggests that, contrary to widely held assumptions of nonspecificity, kinematics is specific to intentions (Jacob and Jeannerod 2005; Kilner et al. 2007a; Naish et al. 2013). The question, however, remains as to whether mirror neuron regions encode intention-specifying information conveyed by visual kinematics.

A major reason for the lack of functional neuroimaging studies addressing this question is the difficulty of identifying patterns of neural response associated with small changes in the detailed spatiotemporal pattern of movement—changes that may even go unnoticed during video presentation. In the present study, this was achieved by combining predictive models of intention discriminability with multivoxel pattern analysis (MVPA). Cavallo et al. (2016) developed a predictive model that isolates the kinematic variables that naïve observers use to discriminate intentions. For this study, we used this model to quantify the degree to which movement patterns encoded intention-information and selected a well-controlled set of movements specifying intentions. Next, we defined an MVPA approach to investigate whether the corresponding intentions could be decoded from action observation brain regions and assess the relative contribution of these regions to classification.

## Methods

### Participants

A total of 21 participants (11 females, mean age = 24.42 years; range = 19–31 years) participated in the current study. All participants were right handed, had normal or correct-to-normal vision, and had no history of neurological or psychological disorder. Written informed consent was obtained from each participant. One participant was excluded from the analysis due to excessive head-motion in the MRI scanner (>3 mm translation, >2° rotation between the sessions). Thus, we report results from 20 participants (10 females, mean age = 24.4 years; range = 19–31 years). The study was approved by the local ethical committee (Comitato di Bioetica d’Ateneo, University of Turin) and was carried out in accordance with the principles of the revised Helsinki Declaration (World Medical Association General Assembly, 2008).

### Stimuli

#### Motion Capture and Video Recording

We employed a dataset of 512 movements obtained by recording 17 naïve participants grasping a bottle with the intent to drink or pour. Apparatus and procedure are described in detail in Cavallo et al. (2016). Briefly, participants’ right hands were outfitted with 20 lightweight retroreflective hemispheric markers (4 mm in diameter). A near-infrared camera motion capture system with 9 cameras (frame rate, 100 Hz; Vicon System) was used to track hand kinematics. Movements were also filmed from a lateral viewpoint using a digital video camera (Sony Handy Cam 3-D, 25 frames/s).

All trials were manually verified for correct marker identification, and passed through a low-pass Butterworth filter with a 6 Hz cutoff. A custom software (Matlab; MathWorks, Natick, MA) was used for data processing and analysis. Kinematics parameters of interest (*n* = 16, see Supplementary Methods) were computed throughout the reach-to-grasp phase of the movement (based on reach onset and grasp offset) at intervals of 10% of the normalized movement time. The second part of the movement, starting from the lift of the bottle, was not considered in the kinematic analysis.

#### Stimuli Selection and Video Editing

**Figure 1.**
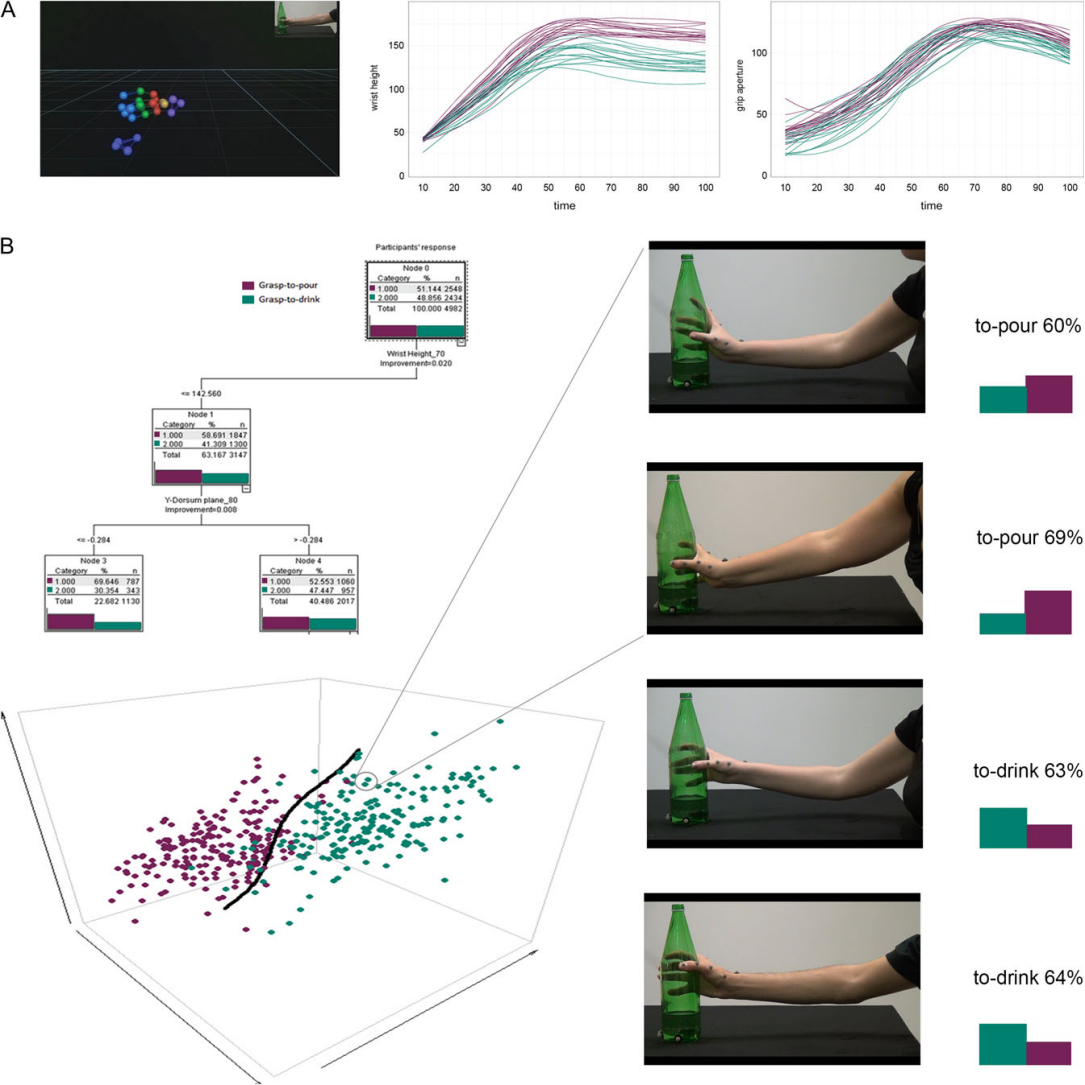
Stimulus selection protocol. (*A*) Video stimulus selection was driven by the content of intention-specific information present in the reach-to-grasp movements. Estimated kinematic features of the movements were input into the CaRT model from Cavallo et al. (2016) in order to generate intention discrimination predictions for each movement (as would be perceived by naïve observers). (*B*) A set of 90 movements (45 grasp-to-pour, 45 grasp-to-drink) for which the CaRT model predicted the highest accuracy was chosen for the current experiment.

Applying Classification and Regression Tree (CaRT) modeling to a battery of action observation experiments, Cavallo et al. (2016) demonstrated that intention discriminability covaries with movement kinematics on a trial-by-trial basis, and relates directly to the expression of discriminant features in the observed movements. In the present analysis, we used the CaRT model generated by Cavallo and colleagues (Cavallo et al. 2016) to quantify intention-specifying information and select a set of 90 movements (45 grasp-to-pour and 45 grasp-to-drink) with a high predicted classification accuracy (0.70 for grasp-to pour movements; 0.70 for grasp-to-drink movements) (Fig. 1).

The corresponding videos were used as stimuli for the fMRI “intention discrimination session.” To ensure that only advance sources of information were made available to participants for judging the agent’s intention, all video clips were temporally occluded at the time the fingers contacted the object using Adobe Premiere Pro CS6 (.mp4 format, disabled audio, 25 frames/s, resolution 1280 × 800 pixel). Thus, each video clip started with the actual reach onset, and ended at grasp offset, with the duration of the video varying according to the actual duration of the movement (from 760 to 1360 ms; see Supplementary Videos S1 and S2).

### Experiment Design and Timing

#### Intention Discrimination Session

**Figure 2.**
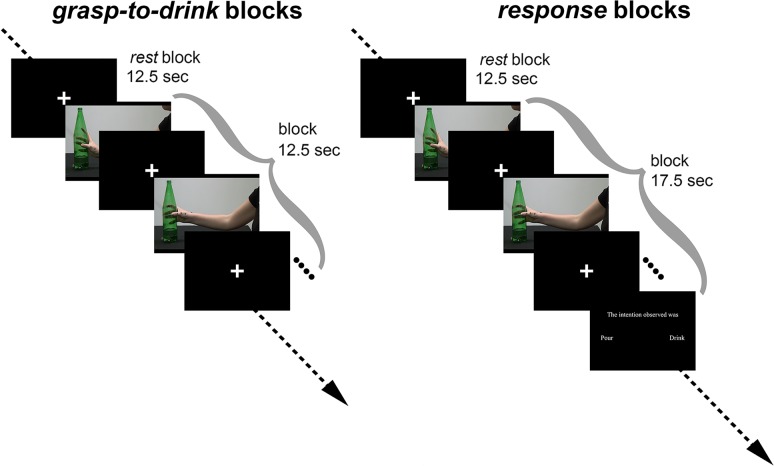
fMRI experimental design for intention discrimination session. The experimental design comprised grasp-to-drink, grasp-to-pour, and response blocks interspersed with rest blocks. A rest block of 12.5 s always preceded the trial sequence for these blocks. Five videos of the same intention (either grasp-to-pour or grasp-to-drink) were presented in succession in grasp-to-drink, grasp-to-pour, and response blocks. An interstimulus interval (ISI) comprised of a white fixation cross at the center of the screen was presented between any 2 of the videos. In addition to the videos, response blocks at the end of the 5 videos requested that participants report the intention of the previously presented set of videos.

During the scanning session, participants completed 3 runs during which they viewed video clips of grasping movements performed with the intent either to pour (grasp-to-pour) or to drink (grasp-to-drink). Each trial started with the static image of the hand for 600 ms, followed by the video of the grasping movement and a compensatory interstimulus interval (white fixation cross) (Fig. 2). The interstimulus interval was set so that each trial lasted 2500 ms. Trials were delivered in blocks, with each block containing 5 videos in a row of the same intention. Participants were instructed to look carefully at each video clip and try to discriminate whether the intention of the observed movement was to drink or to pour. To ensure that participants attended to the video stimuli, in 20% of the trials, after viewing all the video clips in a block, they were also asked to report the intention of the observed movements by means of a button press (“response” block). Participants had 5 s to respond at the end of the response block. “Grasp-to-pour,” “grasp-to-drink,” and “response” blocks were interspersed with 12.5 s rest blocks, where participants fixated to a fixation cross at the center of the screen. Each run in the intention discrimination session was comprised of 12 grasp-to-pour, 12 grasp-to-drink, 6 response, and 31 rest blocks. Three predetermined block sequences (kept constant across subjects) were chosen for the 3 runs. Presented videos in each block were randomly selected from the set of 90 videos.

#### Localizer Session

Subsequent to the main experiment, participants also completed a “localizer session” to functionally determine action observation areas. In the localizer session, participants watched unoccluded videos showing the grasping of a bottle, followed by either pouring some water into a glass or drinking from the bottle. Each trial started with a static image of the hand (300 ms), followed by the video clip of the action sequence (2000 ms) and a white fixation cross (200 ms). Trials were delivered in blocks, with each block containing both reach, grasp, and pour action sequences and reach, grasp, and drink action sequences. The localizer session included 20 blocks displaying action sequences, and 21 rest blocks where participants fixated to a fixation cross at the center of the screen.

### Data Acquisition

High-resolution 3D T1 weighted structural and T2* weighted Echo Planer Images were collected on a whole body Philips Ingenia 3 T MRI scanner, using a 32-channel Philips Sense head coil. The MRI acquisition sequence included a high-resolution structural 3D T1 weighted scan of 180 slices with an in-plane field of view (FOV) of 256 × 256 mm_2_ and 0 mm gap for a resolution of 1 × 1 × 1 mm^3^ (TR = 8.09 ms, TE = 3.70 ms, flip angle = 8°). T2* Gradient-echo (EPI) images sensitive to blood oxygenation level-dependent (BOLD) contrast were used to acquire functional images (45 slices, TR = 2500 ms, in-plane FOV = 240 × 240 mm^2^, resolution = 3 × 3 × 3 mm^3^, TE = 30 ms and flip angle = 90°). A total of 317 volumes per run (for a total of 951) were collected for the intention discrimination session, while 205 volumes were collected for the localizer session.

### Data Analyses

#### Univariate Analysis

Data preprocessing was performed using SPM12 (Wellcome Trust Center for Neuroimaging, University College London; http://www.fil.ion.ucl.ac.uk/spm). For the intention discrimination session, EPI images for different runs were first realigned to the mean image, and then resliced with allowed motion limited to 3 mm translation and 2° rotation within or between runs. Realigned images were then coregistered to the participants’ high-resolution anatomical T1 images. The T1 images were segmented using SPM segmentation function, and normalization parameters to MNI were calculated as deformations. These deformations were then used to normalize resliced functional images. Finally, the normalized images were spatially smoothed using an 8-mm FWHM Gaussian kernel to meet the statistical requirements of the general linear model (GLM).

For both sessions, we defined a GLM separately for each participant. For the localizer session, the model included 2 regressors of interest: action sequence and rest blocks. For the intention discrimination session, the model included 4 regressors of interest: grasp-to-drink, grasp-to-pour, response, and rest blocks. For both sessions, additional regressors of no interest were used to factor out variance due to overall motion calculated during the realignment procedure. All regressors were convolved with a canonical hemodynamic response function. Parameters estimations were filtered using a high pass filter of 128 s to remove low frequency scanner related drifts.

Following the estimation, we first determined a functional action observation localizer based on the localizer session. To investigate group level effects for the localizer session, we entered contrast images of the effects of the regressors of interest for each participant into a random-effects analysis, where we performed the contrast action sequence > rest. The resulting group-level activation map threshold of *P* < 0.001 (uncorrected) served as the action observation localizer.

#### Multivariate Analysis

In this analysis, preprocessing did not include smoothing. Beta images for each run for grasp-to-pour, grasp-to-drink, and rest blocks were used for the classification analysis (Todd et al. 2013). A total of 180 beta images (20 subjects × 3 runs × 3 conditions (grasp-to-pour, grasp-to-drink, rest) were submitted to the MVPA analysis. We merged beta files for each run and from all subjects into 4D nifti images (three 4D files with 3 conditions for each subject). Further, we *z*-normalized and averaged our data per subject per condition for a total of 60 samples. Averaging was performed to decrease intra-subject variability and increase the signal to noise ratio (Quadflieg et al. 2011; Ghio et al. 2016).

We selected the voxels to be used for classification by generating a mask between action observation areas (as identified by the localizer) and the regions of interest (ROIs) defined by an automatic anatomic labeling toolbox (Tzourio-Mazoyer et al. 2002). Using this mask, we then extracted the beta values for the 2 intentions from the images in intention discrimination session. Feature selection was performed using ANOVA to select voxels whose activation was modulated across the 2 classes (*P* < 0.05). The ANOVA was performed independently on the training dataset to avoid biasing the classifier. A linear support vector machine (SVM) classifier model (Vapnik 1995) was used to classify to-pour and to-drink intentions.

We used a leave-one-subject-out cross-validation procedure, excluding one participant at each iteration (test set) and using the other participants as the training set. This procedure was repeated until data from all subjects were utilized as test set. Accuracy values from each iteration were averaged to obtain classification scores for each ROI. Statistical significance of these results was estimated based on permutation testing (10 000 simulations). All MVPA data analysis was performed with PyMVPA (Hanke et al. 2009).

## Results

### Observers are Able to Classify Intention Just Using the Available Kinematic Information

Response accuracy in response blocks was significantly above the 0.50 chance level for both grasp-to-drink (mean ± standard error [SE] = 0.74 ± 0.04; *t*_19_ = 5.57, *P* < 0.001; 95% CI = 0.65–0.83) and grasp-to-pour movements (mean ± SE = 0.73 ± 0.05; *t*_19_ = 4.24, *P* < 0.001; 95% CI = 0.62–0.84). Participant response accuracies did not differ from those predicted by the CaRT model (grasp-to-drink: *t*_19_ = 0.81; *P* = 0.43; grasp-to-pour: *t*_19_ = 0.59; *P* = 0.56). In confirming the predictive accuracy of the model, this analysis indicates that participants were able to pick up intention-specifying information conveyed by slight variations in movement kinematics.

### Observation of Grasp-to-Pour and Grasp-to-Drink Movements Activates the Frontoparietal Nodes of the Action Observation Network

**Figure 3.**
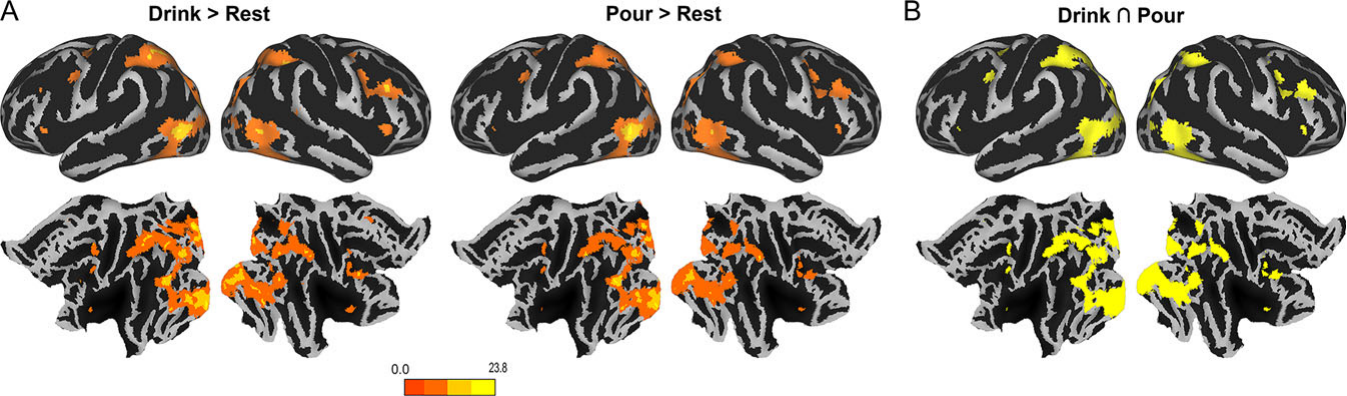
Brain activations during observation of reach-to-grasp movements. (*A*) Frontoparietal activation during observation of grasp-to-drink and grasp-to-pour, compared with rest. (*B*) A conjunction map of brain regions commonly activated by the observation of grasp-to-drink and grasp-to-pour movements highlights common frontal, parietal, as well as visual brain regions.

We next sought to identify the neural mechanisms underlying the ability to process this information. To probe the involvement of putative mirror neuron regions, we first entered linear contrasts of regression coefficients, computed for each participant into a random-effects analysis. Figure 3*A* shows regions of significant activation in the univariate comparisons grasp-to-pour > rest, and grasp-to-drink > rest. As revealed by a conjunction analysis (Fig. 3*B*), areas commonly activated by the viewing of grasp-to-pour and grasp-to-drink movements included the bilateral inferior parietal lobule (IPL), the bilateral superior parietal lobule (SPL), and the bilateral inferior frontal gyrus (IFG). Additionally, we found bilateral activations in the mid frontal gyrus (MFG), the precentral gyrus, and the occipital cortex, extending from the calcarine sulcus to the inferior occipital cortex (see Supplementary Table S1). Contrasting grasp-to-pour and grasp-to-drink movements revealed no differential activation in the action observation network, or any other region.

### Intention-Specific Information can be Decoded From Action Observation Regions

In order to investigate whether the intention of the observed act could be decoded from action observation regions, we next applied MVPA to spatial patterns of brain responses under the 2 possible intentions. We found that several regions predicted the intention of the observed motor act (Table 1). The highest classification accuracy within the parietofrontal action observation network was achieved in IPL (accuracy = 0.78, *P* < 0.001). Decoding accuracies in SPL (accuracy = 0.73, *P* < 0.01), IFG (accuracy = 0.68, *P* < 0.05), and MFG (accuracy = 0.65, *P* < 0.05) were also significantly above chance level.

**Table 1:** Classification scores for action observation regions.

Brain region	Classification score	Permutation *P*-values	AUC
Inferior parietal lobule	0.78^*^	<0.001	0.85
Inferior frontal gyrus	0.68^*^	<0.05	0.70
Superior parietal lobule	0.73^*^	<0.01	0.85
Left calcarine sulcus	0.78^*^	<0.01	0.90
Mid frontal gyrus	0.65^*^	<0.05	0.70
Right inferior occipital gyrus	0.3	0.999	0.25
Left mid occipital	0.53	0.428	0.65
Precentral gyrus	0.60	0.091	0.60
Superior temporal gyrus	0.45	0.818	0.40
Mid temporal gyrus	0.55	0.271	0.70
Inferior temporal gyrus	0.48	0.686	0.35
Supplementary motor area	0.58	0.190	0.55
Premotor	0.6	0.102	0.40

^*^Significant classification based on a permutation testing.

### Distinct Contributions of Action Observation Regions Towards Intention Classification

Having established that several regions within the action observation network predict the intention of the observed motor act, we next attempted to characterize the relative contribution of these regions to intention classification.

**Figure 4.**
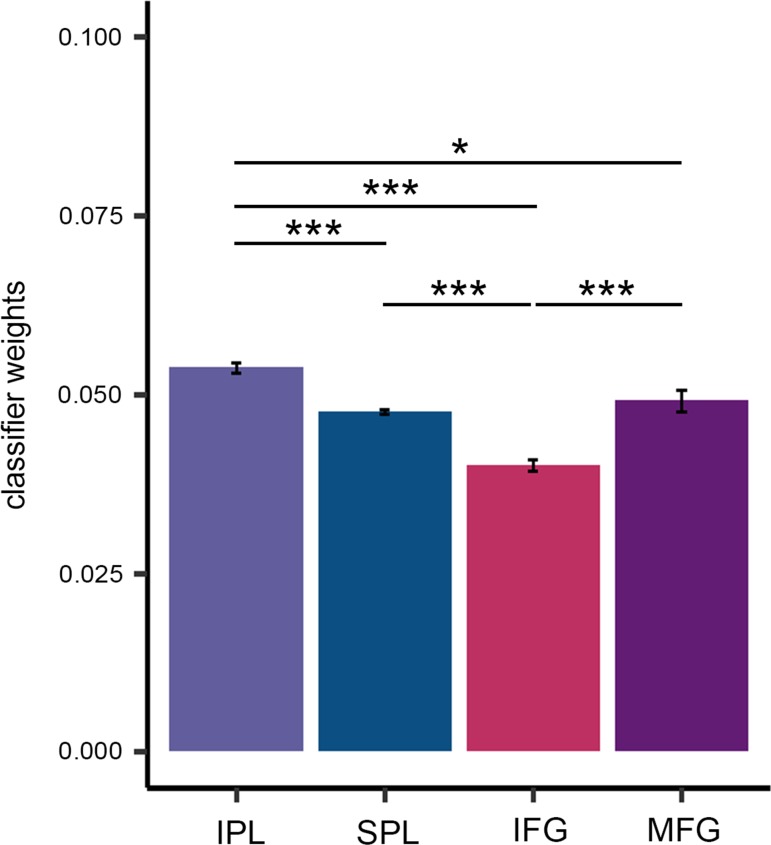
Differential contribution of frontoparietal nodes in action observation network towards intention classification. Averaged absolute classifier importance values across the 4 regions. Error bars represent standard error of mean (SEM). Asterisks indicate significant differences between brain regions (*P < 0.05; ***P < 0.001).

One method to accomplish this is to perform an SVM classification on a combined voxel set including IPL, SPL, IFG, and MFG and then extract the weight parameters resulting from the classifier. The absolute value of a voxel weight reflects the contribution of that voxel to the discrimination process in the context of the other voxels included in the classification analysis (Mahmoudi et al. 2012; Hebart and Baker 2017). A heuristic approach to determine the contribution of a region of interest to the discrimination process is thus to compute the average of absolute value of voxels weights obtained at each iteration of the cross-validation procedure in that specific region (Pereira et al. 2009). An analysis of variance (ANOVA) conducted on this measure yielded a significant main effect of brain region (*F*_(3,57)_ = 41.36, *P* < 0.001, partial *η*^2^ = 0.69). Pairwise comparisons, Holm–Bonferroni corrected (Holm 1979), revealed that classifier weights were significantly higher in IPL compared with SPL (*P* < 0.001), IFG (*P* < 0.001), and MFG (*P* < 0.02) (Fig. 4). Classifier weights were also higher in SPL compared with IFG (*P* < 0.001) and in MFG compared with IFG (*P* < 0.001). No difference was observed between SPL and MFG (*P* = 0.338).

### Consistency in Spatial Distribution of Voxels Selected for Classification is Similar Across Action Observation Regions

**Figure 5.**
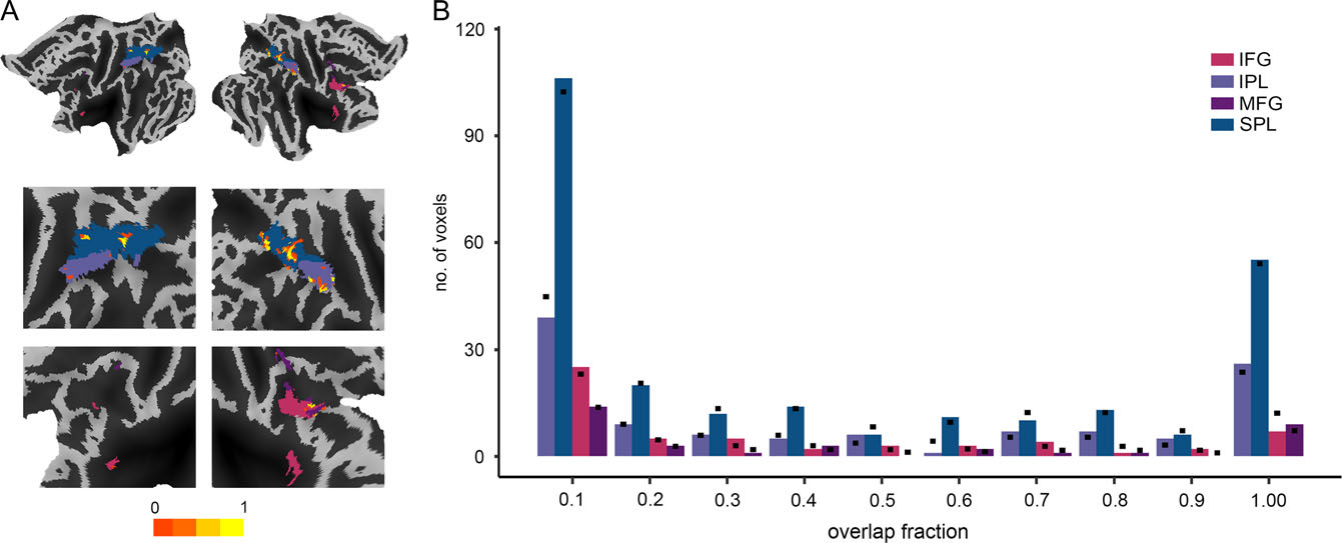
Overlap fractions of voxels selected for classification in action observation network. (*A*) Overlap fraction maps for top 5% voxels selected for classification. (*B*) Bar graph representing consistency in the spatial distribution of voxels used for classification in IPL, SPL, IFG, and MFG. Higher fractions correspond to a consistent selection of the same voxels over participants. Black dots represent expected number of voxels from a given region for a given overlap fraction.

Voxels selected for the classification may be either the same or different across subjects. To assess the consistency in spatial distribution of selected voxels across subjects, we generated an overlap fraction map, assigning each voxel in the combined voxel set a value between 0 and 1. A value of 1 would indicate that the voxel was always among the top 5% voxels selected for classification while a value of 0 would indicate that it was never among the selected voxels. Comparison between the ROIs revealed no significant difference in overlap fractions (*χ*^2^ = 20.43, *P* = 0.81) (Fig. 5). This suggests that the consistency in the spatial distribution of voxels used for classification was similar across action observation regions.

## Discussion

A longstanding debate has endured on the possibility of understanding the intentions of other persons through observation of their actions (Jacob and Jeannerod 2005). The debate continues on whether intentions lead to specific kinematic patterns from which observers may obtain information about others’ mental states (Kilner et al. 2007b; Ansuini et al. 2015). Additionally, it is unclear whether action processing and intention understanding are centered in classical mirror neuron regions, or whether they require more than merely the mirror neuron system (Kilner 2011; Becchio et al. 2012; Kilner and Lemon 2013).

In the present study, we combined experiments designed according to rigorous kinematic techniques with MVPA of neuroimaging data to examine whether intention-specifying information conveyed by visual kinematics can be read-out from action observation regions. Participants were exposed to temporally occluded grasp-to-pour and grasp-to-drink movements identical except for subtle differences in movement kinematics. We found that, besides visual areas, regions considered to be part of the human mirror neuron system carried discriminative information about the intentions of the observed acts.

Evidence that cortical action representations are tuned to movement kinematics has been provided by studies using fMRI (Dayan et al. 2007; Casile et al. 2010), magnetoencephalography (MEG) (Press et al. 2011), electroencephalography (EEG) (Avanzini et al. 2012), and transcranial magnetic stimulation (TMS) (Agosta et al. 2016). For example, Dayan et al. (2007) using fMRI found that kinematic invariants differentially activated a widespread network of areas subserving both action execution and action observation functions.

The central advance of the present study is the demonstration that coding of subtle variations in movement kinematics within these areas provides access to the intention of the agent performing the observed motor act. This advanced information pickup from observed movement patterns is not captured by existing models of the mirror neuron function (Kilner et al. 2007a; Chersi et al. 2011; Kilner 2011); for review, see Giese and Rizzolatti 2015), which typically make the simplifying assumption that small changes in visual kinematics do not contribute to action representation in putative mirror neuron regions. For example, “motor chains” models assume that an “identical motor act” (e.g., grasping a bottle) is chained to different subsequent acts based on the “context” of the observed action (Chersi et al. 2011). The presence of an empty glass close to the bottle, for example, may lead to the selection of a neuronal chain linking grasping to pouring. On the other hand, if an ice bucket were close to the bottle, then grasping would most probably be linked to placing. On this account, only the processing of contextual cues, which cannot be achieved by mirror neuron activity, may enable the observer to select the appropriate motor chain (Jacob 2013).

Our results demonstrate that, contrary to this assumption, even in absence of contextual cues, putative mirror neuron areas within the action observation network carry intention-specific kinematic information. While this does not rule out the significance of context, it opens the possibility that, in the absence of discriminative contextual information, the kinematic features of the observed act lead to the activation of the most appropriate neuronal chain.

In our study, classifier weights were higher in IPL. This is consistent with human neuroimaging studies assigning IPL a prominent role in coding of intention (Hamilton and Grafton 2008; Jastorff et al. 2010; Oosterhof et al. 2010). In monkeys, IPL has been shown to contain a higher proportion of motor neurons whose response is modulated by the intention of the executed motor act, in comparison to IFG (Bonini et al. 2010). The proportion of mirror neurons tuned to intention, however, does not appear to differ between the 2 regions during action observation (Bonini et al. 2010), suggesting that in monkeys IPL and IFG contribute similarly to coding of others’ intentions. This could suggest that the functional specificity of IPL is more pronounced in humans compared with nonhuman primates. An important goal for future studies will be to track the temporal dynamics of intention encoding across different action observation regions in order to understand how they relate to the pick-up of intention specifying information (Ortigue et al. 2010; Perry et al. 2018).

From a theoretical perspective, these results provide insights into conceptual questions regarding action mirroring: do we mirror movements or intentions? (Csibra 1993; Hasson and Frith 2016). It has been proposed that the extension of mirror neurons to the domain of intentions generates a tension between the specificity of the observed movement and the intention associated with that act. The tension is supposed to arise from the fact that the more mirroring is narrowly tuned to a specific motor act (i.e., mirrors movement), the less evidence it provides for intention understanding; the more mirroring is broadly tuned to a goal (i.e., mirrors intentions), the less evidence it provides for matching the specific motor act (Hasson and Frith 2016). In other words, one cannot argue at once that mirror neurons in the observer’s brain codes both movements and intentions (Jacob 2013). Our findings argue against this view. If intentions translate into slight kinematic variations, then mirroring these variations may indeed be crucial to perceive the agent’s intention.

## Supplementary Material

Supplementary Data
