# Supplementary material for: Action Observation Areas Represent Intentions From Subtle Kinematic Features
Source: Cereb Cortex. 2018 May 2;28(7):2647–54. doi: 10.1093/cercor/bhy098 (PMC5998953; doi:10.1093/cercor/bhy098)
Supplement: Supplementary Data [file bhy098_suppl1.zip › bhy098Supplementary_Methods.docx]

**S1 Methods**

**Estimated movement kinematics features** **of interest**

We used a custom software (Matlab; MathWorks, Natick, MA) to compute two sets of parameters of interest: F_global_  and F_local_ parameters. F_global_ parameters were expressed with respect to the global frame of reference, i.e., the frame of reference of the motion capture system. Within this frame of reference, we computed the following parameters:

1. Wrist Velocity, defined as the module of the velocity of the wrist marker (mm/sec);
2. Wrist Height, defined as the z-component of the wrist marker (mm);
3. Wrist Horizontal Trajectory, defined as the x-component of the wrist marker (mm);
4. Grip Aperture, defined as the distance between the marker placed on thumb tip and the one placed on the tip of the index finger (mm).

To provide a better characterization of the hand joint movements, the second set of parameters was expressed with respect to a local frame of reference centered on the hand (i.e., F_local_; see (Ansuini et al. 2015) for a detailed description of the F_local_).Within F_local_ we computed the following parameters:

1. x-, y-, and z-thumb defined as x-, y- and z-coordinates for the thumb with respect to F_local_ (mm);
2. x-, y-, and z-index defined as x-, y- and z-coordinates for the index with respect to F_local_ (mm);
3. x-, y-, and z-finger plane defined as x-, y- and z-components of the thumb-index plane, i.e., the three-dimensional components of the vector that is orthogonal to the plane, providing information about the abduction/adduction movement of the thumb and index finger irrespective of the effects of wrist rotation and of finger flexion/extension;
4. x-, y-, and z-dorsum plane defined as x-, y- and z-components of the radius-phalanx plane, providing information about the abduction, adduction and rotation of the hand dorsum irrespective of the effects of wrist rotation.

**References**

Ansuini C, Cavallo A, Koul A, Jacono M, Yang Y, Becchio C. 2015. Predicting object size from hand kinematics: a temporal perspective. PLoS One. 10:e0120432.
