# Supplementary material for: Action Observation Areas Represent Intentions From Subtle Kinematic Features
Source: Cereb Cortex. 2018 May 2;28(7):2647–54. doi: 10.1093/cercor/bhy098 (PMC5998953; doi:10.1093/cercor/bhy098)
Supplement: Supplementary Data [file bhy098_suppl1.zip › bhy098Supplementary_Table_S1.docx]

Table S1. Peak MNI coordinates for *grasp-to-drink>rest* and *grasp-to-pour>rest* contrasts.

| **Anatomical Area** | ***grasp-to-drink>rest*** | | | | ***grasp-to-pour>rest*** | | | |
| --- | --- | --- | --- | --- | --- | --- | --- | --- |
|  | **t-scores** | **Peak MNI coordinates** | | | **t-scores** | **Peak MNI coordinates** | | |
|  |  | x | y | z |  | x | y | Z |
| **Occipital Lobe** |  |  |  |  |  |  |  |  |
| Left Calcarine Sulcus | 28.98 | -6 | -72 | 10 | 25.23 | -6 | -70 | 10 |
| Left Mid Occipital | 18.62 | -20 | -88 | 18 | 20.74 | -44 | -72 | 4 |
| Right Inferior Occipital | 17.32 | 36 | -64 | -10 | 16.21 | 36 | -64 | -10 |
|  |  |  |  |  |  |  |  |  |
| **Parietal Lobe** |  |  |  |  |  |  |  |  |
| Left Superior Parietal | 14.20 | -26 | -60 | 58 | 11.50 | -32 | -54 | 62 |
| Right Inferior Parietal | 13.99 | 40 | -46 | 52 | 13.92 | 42 | -44 | 50 |
| Left Inferior Parietal | 13.38 | -36 | -52 | 56 | 13.47 | -42 | -42 | 48 |
| Right Superior Parietal | 9.27 | 18 | -66 | 58 |  |  |  |  |
|  |  |  |  |  |  |  |  |  |
| **Frontal Lobe** |  |  |  |  |  |  |  |  |
| Right Inferior Frontal | 14.36 | 46 | 28 | 26 | 11.41 | 48 | 28 | 22 |
| Left Precentral | 12.78 | -42 | 0 | 36 | 12.14 | -44 | -4 | 34 |
| Left Superior Frontal | 12.26 | -28 | -8 | 60 |  |  |  |  |
| Right Mid Frontal | 10.90 | 44 | 36 | 20 | 10.49 | 44 | 36 | 19 |
| Right Orbito-frontal | 9.52 | 40 | 44 | -14 |  |  |  |  |
| Right Precentral | 9.50 | 44 | 6 | 34 | 8.03 | 42 | 2 | 44 |
| Left Supplementary Area | 8.86 | -2 | 8 | 52 | 8.11 | -4 | 8 | 50 |
| Right Supplementary Area | 8.45 | 4 | 14 | 52 | 6.99 | 6 | 16 | 50 |
| Left Inferior Frontal | 8.23 | -54 | 10 | 26 | 7.21 | -54 | 10 | 26 |
| Left Mid Frontal | 7.38 | -44 | 52 | -4 | 11.39 | -46 | -62 | 2 |
|  |  |  |  |  |  |  |  |  |
| **Temporal Lobe** |  |  |  |  |  |  |  |  |
| Right Mid Temporal | 13.17 | 46 | -66 | 2 | 12.62 | 48 | -68 | 0 |
| Left Mid Temporal Gyrus | 11.23 | -46 | -58 | 2 | 11.39 | -46 | -62 | 2 |
| Right Superior Temporal | 7.76 | 62 | -36 | 16 |  |  |  |  |
|  |  |  |  |  |  |  |  |  |
| **Sub-cortical regions** |  |  |  |  |  |  |  |  |
| Left Thalamus | 14.52 | -16 | -28 | 2 | 10.39 | -18 | -30 | 8 |
| Right Pallidum | 7.94 | 18 | -2 | 2 |  |  |  |  |
|  |  |  |  |  |  |  |  |  |
| **Cerebellum** |  |  |  |  |  |  |  |  |
| Left Cerebellum | 7.87 | -22 | -42 | -44 | 8.56 | -22 | -42 | -44 |
| Right Cerebellum | 7.27 | 22 | -46 | -44 | 12.59 | 10 | -74 | -24 |
| Vermis |  |  |  |  | 7.62 | 0 | -58 | -36 |
|  |  |  |  |  |  |  |  |  |
| **Insula** |  |  |  |  |  |  |  |  |
| Left Insula | 8.69 | -30 | 24 | -2 | 7.22 | -30 | 24 | -2 |

*Activation peaks that passed a voxel-wise FWE threshold p < 0.05 are reported.*
